# Developing a competency framework for artificial intelligence in undergraduate dental education

**DOI:** 10.3389/fdmed.2026.1849000

**Published:** 2026-06-11

**Authors:** Supachai Chuenjitwongsa, Tanit Arunratanothai, Dinesh Rokaya, Lakshman P. Samaranayake, Anjalee Vacharaksa, Thanaphum Osathanon

**Affiliations:** 1 Dental Education Unit, Office of Academic Affairs, Faculty of Dentistry, Chulalongkorn University, Bangkok, Thailand; 2 Clinical Sciences Department, College of Dentistry, Ajman University, Ajman, United Arab Emirates; 3 Center of Medical and Bio-Allied Health Sciences Research, Ajman University, Ajman, United Arab Emirates; 4 Global Research Cell, Dr. D. Y. Patil Dental College and Hospital, Dr. D. Y. Patil Vidyapeeth, Pimpri, Pune, India; 5 Faculty of Dentistry, The University of Hong Kong, Hong Kong, Special Administrative Region; 6 Center of Artificial Intelligence and Innovation, Faculty of Dentistry, Chulalongkorn University, Bangkok, Thailand; 7 Master of Science Program in Geriatric Dentistry and Special Care Dentistry, Faculty of Dentistry, Chulalongkorn University, Bangkok, Thailand; 8 Department of Microbiology, Faculty of Dentistry, Chulalongkorn University, Bangkok, Thailand; 9 Research Unit on Oral Microbiology and Immunology, Faculty of Dentistry, Chulalongkorn University, Bangkok, Thailand

**Keywords:** artificial intelligence, clinical integration, competency framework, dental education, ethical considerations

## Abstract

Artificial intelligence (AI) technologies are evolving rapidly and are increasingly being adopted across various domains, including dentistry. Therefore, the standardised integration of AI into the dental curriculum is crucial to cultivate students’ knowledge, skills, attitudes, and awareness for effective engagement within an increasingly AI-driven educational and clinical ecosystem. To achieve these learning outcomes, a well-structured competency framework is required to outline the specific AI-related competencies expected of dental students. This article presents the development of such a competency framework, focusing on three key domains: i) AI fundamentals, ii) AI implementation, and iii) ethical and professional conduct in AI use. Particular emphasis is placed on the humane dimensions in AI integration, highlighting responsibility, accountability, and ethical considerations. The framework draws on the essential components of AI literacy promulgated by a dental school in Thailand and aligns with similar guidelines from other authoritative international sources. The examples of topic structures and approaches for integrating these competencies into the undergraduate curriculum are also discussed. Ultimately, this competency framework seeks to strengthen AI literacy and prepare a new generation of dental professionals to thrive in the emerging landscape of digital transformation and AI-driven education.

## 1 Introduction

Artificial intelligence (AI) technologies are transforming the landscape of various fields, especially education and healthcare. A range of AI-based applications have been developed and validated in dental practice settings, including automated image detection for clinical and radiographic evaluation, virtual treatment planning, intelligent clinical management, and adaptive patient education. Beyond clinical applications, AI is increasingly influential in dental education. It supports curriculum design and development, teaching delivery, skills assessment, and research and innovation (1, 2). These developments highlight both the opportunities and challenges posed by AI's growing role in dentistry. To fully harness its benefits while maintaining professional and ethical integrity, educators and healthcare providers must adapt their strategies and cultivate AI literacy. The present article aims to provide perspectives on the development of an AI competency framework within the undergraduate dental curriculum and describes its strategic implementation in both curricular and extracurricular contexts using the example of one dental school in Thailand.

## 2 An organisational strategy for AI

The integration of AI in healthcare organisations offers immense potential but also faces significant systemic challenges, including misalignment of institutional procedures, data security concerns, funding limitations, and gaps in regulatory frameworks (3). To ensure responsible and sustainable adoption, institutions must establish comprehensive governance frameworks for AI across domains such as education, research, procurement, data management, and clinical operations (3). To effectively implement AI within an organisation, it is imperative to recognise the significant role that explicit regulations play in fostering trust, ensuring safety, and directing ethical application (4). To enhance AI competence, the school should outline strategies, including establishing an AI competency framework, conducting hands-on workshops, and providing practical training to ensure that faculty members, students, and staff can utilise AI efficiently.

Additionally, the strategy emphasises the integration of AI in ways aligned with ethical guidelines by developing practical, ethical usage frameworks and awareness campaigns, fostering responsible AI practices across all areas of the faculty's activities. It focuses on empowering faculty, staff, and students to effectively use AI to improve efficiency, learning, and patient outcomes.

Key initiatives include the creation of an AI competency framework, hands-on workshops, and practical training opportunities. Ethical integration is emphasised through the development of responsible use guidelines and awareness campaigns. The goal is to foster an institutional culture of trustworthy, human-centred AI practice. It focuses on empowering faculty, staff, and students to effectively use AI to improve efficiency, learning, and patient outcomes.

Another key focus of the strategy is the development of AI systems tailored to the faculty's needs in work, education, research, and services. This includes providing targeted research funding to create AI systems and establishing collaborations with external organisations to drive innovative AI solutions that address real-world institutional challenges. Together, these efforts aim to establish a robust and ethical AI ecosystem that supports its mission of academic and professional excellence.

In accordance with this strategy, it has promulgated guiding principles for the use of AI technologies as follows: AI technologies should be integrated responsibly into teaching, research, and clinical activities to enhance, rather than replace, human expertise.

**Figure 1:**
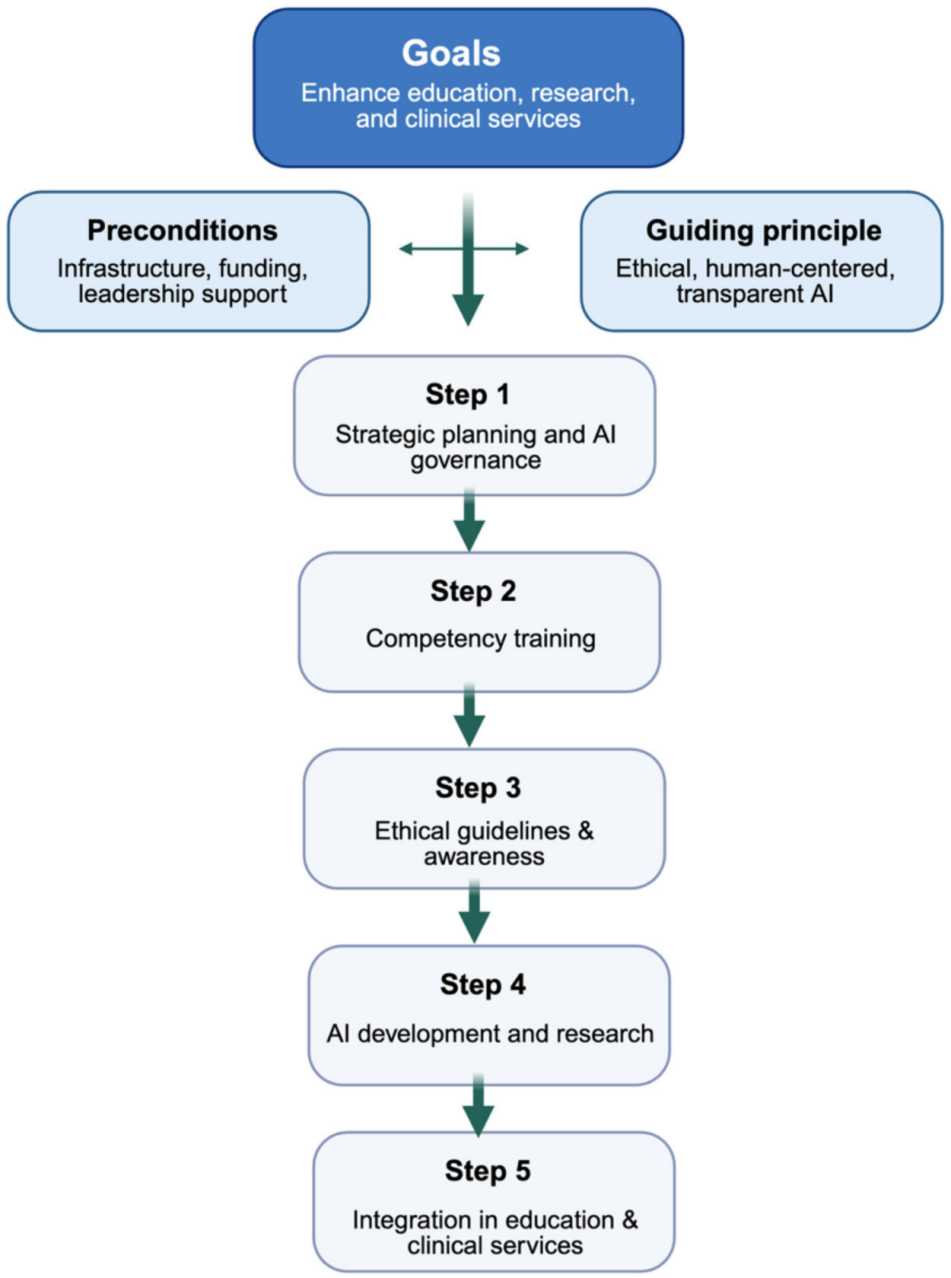
Use case flow diagram illustrating the strategic integration of AI. Created in BioRender. https://BioRender.com/bx2eo7d.

Figure 1 presents the flow, outlining key components: stakeholder involvement (Actors), institutional objectives (Goal), foundational requirements (Preconditions), and phased implementation steps—from strategic planning to capacity building, ethical integration, system development, and practical deployment. The process culminates in measurable outcomes and is guided by principles of ethical, human-centred AI. In recent years, the transformative power of artificial intelligence (AI) has become increasingly evident across healthcare and education. However, the path to integrating AI into complex institutions—such as dental schools—requires more than just technology. It demands vision, planning, ethical foresight, and capacity building.

The strategic implementation in Faculty of Dentistry, Chulalongkorn University is described as the example. This vision took shape as part of a broader digital transformation initiative. Recognising the potential of AI to enhance learning, research, and patient care, the Faculty embarked on a journey to build a sustainable, human-centred AI ecosystem. The first step was to identify the key actors involved in the transformation. The collaboration and buy-in were critical to success. To enhance education, research, and clinical services through AI, the Faculty set out to design a structured, strategic approach. But before implementation, certain preconditions had to be met: a supportive digital infrastructure, institutional funding, and leadership commitment to ethical innovation. The Faculty initiated its strategy with strategic planning and the development of AI governance frameworks. These would ensure that AI integration aligned with institutional values and complied with ethical and regulatory standards.

Next came capacity building. An AI competency framework was introduced, supported by workshops and training sessions to equip faculty and students with the skills and confidence needed to use AI tools effectively. This step was crucial in creating a culture of innovation that extended beyond technology into teaching and daily operations. Understanding the ethical risks associated with AI, the Faculty placed strong emphasis on responsible AI use. Awareness campaigns, discussions, and the development of internal guidelines ensured that AI would serve as a tool to enhance, not replace, human expertise. To support innovation, the Faculty also invested in developing AI systems. By allocating research funding and fostering partnerships with external AI organisations, the school aimed to create custom solutions tailored to dental education and clinical needs. With these foundations in place, the Faculty began to integrate AI into its core activities. The Faculty observed improved learning outcomes and greater research efficiency. Just as importantly, a culture of ethical, transparent, and human-centred AI began to take root.

The Faculty's guiding principles remained clear throughout: AI should be integrated responsibly, used to enhance, not replace, human expertise, and implemented with a commitment to transparency, inclusivity, and ongoing education. This use case demonstrates how a thoughtful, institution-wide strategy can turn the promise of AI into practical progress—one that empowers people and respects ethics. It advances the mission of academic and clinical excellence.

## 3 AI competency framework alignment within an institutional setting: guiding principles

The following is an outline of the guiding principles for aligning an AI competency framework within an institutional setting, categorised into several key domains.

### 3.1 General domain

When utilising AI technologies, it is crucial to explicitly reference and define the scope of their application within the work context. Technologies are intended as tools to support users’ work; however, users remain ultimately responsible for data accuracy and outcomes produced. The adoption of AI technologies must adhere to relevant ethical principles, regulations, and legal frameworks, prioritising a human-centric approach and ensuring the logical suitability of their application.

### 3.2 Teaching domain

Educators must possess a comprehensive understanding of the capabilities and limitations of the AI technologies they choose to integrate and should design teaching and assessment methodologies aligned with these tools’ capabilities. Educators should explicitly outline in the course syllabus the scope and guidelines for using and integrating AI technologies and explain the rationale to students.

### 3.3 Research domain

Researchers should comprehensively understand the potential and limitations of the AI technologies deployed at different stages of the research processes. The researcher must declare and document the use of AI technologies at each stage of the research process. Researchers must review and validate the data and results generated by AI technologies, ensuring their accuracy and relevance. Researchers must ensure that the use of AI technologies in research is in accordance with ethical research principles.

### 3.4 Clinical-related domain

Clinicians should regard AI technologies as supplementary and supportive tools in clinical practice, ensuring they complement, rather than replace, clinical judgment. Clinicians must verify and interpret AI-generated outputs within the context of professional expertise and patient care standards.

### 3.5 Confidentiality, privacy, and data protection domain

Users must ensure that no confidential or personal data is input into AI systems beyond the organisation without the explicit consent of the relevant individuals. All use of AI must comply with applicable laws, ethical standards, and institutional policies on data privacy and confidentiality.

In conclusion, the development of the Faculty’s AI competency framework must align with these principles to ensure consistent, ethical, and effective integration of AI technologies across all domains of activity.

## 4 Identifying Key competencies for AI in dental education

To establish the competency framework, a multifaceted approach was undertaken, involving a comprehensive literature review, alignment with existing guidelines on AI literacy and ethical use, and expert consultations. The key competencies identified fell under three domains: AI Fundamentals, AI Implementation, and Code of Conduct for AI Use.

### 4.1 AI landscape in dental education

A comprehensive literature review was conducted to explore the current landscape of AI utilisation in dental education. The literature was searched in the PubMed and Scopus databases to identify relevant information on AI in dental education. The search term was shown in Supplementary Table 1. This article focuses on the process of developing AI competencies. Therefore, an extensive and detailed discussion on the literature review and the current landscape was not included.

A core educational curriculum for dental students to achieve AI literacy has been proposed in 4 domains: knowledge, use cases, evaluation matrices, and its limited generalizability (5). However, concerns regarding the ethical and legal implications of AI implementation are underscored, emphasising the need for a consensus on the safe and responsible use of AI in dental healthcare (2). Taken together, AI in dental education focuses on enhancing clinical skills by providing innovative teaching and learning approaches, improving academic performance by predicting students’ achievement and forming personalised learning schemes, enhancing assessment by providing formative feedback, and improving educational delivery by integrating AI into the curriculum.

### 4.2 Alignment with the existing guideline

Guidelines for the ethical and literary use of AI were identified. The documents employed for evaluation are the UNESCO AI competency framework for teachers, UNESCO AI competency framework for students, WHO Ethics and Governance of Artificial Intelligence for Health, National Artificial Intelligence Action Plan for Thailand Development 2022–2027, FDI White Paper Artificial Intelligence for Dentistry, and Chulalongkorn University General AI Competency Framework (6–9). These documents encapsulate the global perspective on AI competency across the educational and healthcare sectors at both local and international levels.

The White Paper on “Artificial Intelligence for Dentistry” by the FDI Artificial Intelligence Working Group highlights the transformative potential of AI in dentistry, covering the integration in individual care, community health (10, 11). These articles focus on ethical concerns, governance strategies, and regulations ensuring the safe and responsible use of AI. Similarly, the WHO Ethics and Governance of Artificial Intelligence for Health emphasises that the integration of AI technologies must adhere to principles of privacy, transparency, human agency, and public health benefits. The UNECSO AI Competency framework for Students conceptualises the skills and knowledge required for students to thrive in an increasingly AI-driven world, including the ability to recognise and question AI, the ethical and responsible use of AI, and the leveraging of AI for personal and social benefits. The alignment with these guidelines contributed to the development of the AI competency framework, ensuring that the identified competencies align with international best practices and address key ethical and governance considerations.

### 4.3 Expert consultations

The competency framework is consulted with experts through an informal discussion. The participating experts are two dental educators, one computational engineer, and one AI expert. These experts provided input to reshape the AI competency framework to align with the multifaceted components of dental school: education, research, and healthcare services. The expert inputs covered the needs, gaps and challenges that dental students and faculty may face in adopting AI in their practices.

### 4.4 Adoption of the Chulalongkorn University general AI competency framework (chula AI 10)

Aligned with the General AI Competency Framework from Chulalongkorn University, the dental curriculum expands its AI competencies to foster student proficiency in applying AI tools responsibly and creatively, supported by strong ethical foundations and evidence-based practice. The proposed AI framework for the dental curriculum, aligned with the general AI competency framework, is shown in Table 1.

**Table 1:** AI competency framework for the dental curriculum based on chulalongkorn university general AI competency framework (CU-AI 10) and their alignment with reference documents.

Chulalongkorn General AI Competency Framework (CUAI)	AI Competency Framework for the Dental Curriculum	UNESCO – AI Competency Framework for Students (2024)	FDI White Paper – Artificial Intelligence for Dentistry (2024)	Ethics and governance of artificial intelligence for health: WHO guidance (Thai edition)	National Artificial Intelligence Action Plan for Thailand’s Development (2022–2027)
1. AI knowledge					
CUAI-1: Foundational AI Competency Demonstrate foundational knowledge of AI and Machine Learning, including their evolving capabilities, limitations, and the related digital skills needed to effectively engage with these technologies.	1. Demonstrates essential digital skills for effective interaction with AI technologies. 2. Possesses a comprehensive understanding of artificial intelligence and machine learning principles. 3. Recognizes the progressive capabilities of AI systems as well as their inherent limitations	Aspect: AI techniques and applications: AI foundations (Level 1: Understand) “Students are expected to build basic knowledge and skills on AI, particularly with respect to data and algorithms”	Section 1: Background and Objectives Defines AI and the general capabilities of AI in healthcare and dentistry. Section 4: Education and the workforce. “A major challenge to be highlighted is that the current dental workforce shows only limited digital literacy.” “Choosing between different AI tools for a specific practice, commissioning AI for community and public health purposes, and appraising AI with regards to its capacity and quality will require knowledge about the way AI works.” “The pitfalls around datasets underlying an AI application, but also around measuring the performance of an AI application must be known.”	Chapter 5 – Introduction to the six ethical principles for AI for health (p.77) Establishes foundational understanding that AI is a set of computational techniques whose use must be informed by ethics, capabilities and limits. (p.77–78)	Strategy 3: Increasing personnel capacity and developing AI education
CUAI-2: AI Use Case Evaluation Assess the feasibility, suitability, and ethical implications of using AI in various situations, considering its benefits, risks, biases, limitations, and costs.	1. Demonstrates the ability to assess AI applications across diverse domains (e.g., education, research, healthcare). 2. Evaluates feasibility, appropriateness, and ethical implications of implementing AI for specific tasks. 3. Analyzes advantages, risks, inherent biases, limitations, and financial consequences of AI tools to support informed decision-making	Aspect: AI techniques and applications: Application skills (Level 2: Apply) and Ethics of AI: Embodied ethics/Safe and responsible use “Students are expected to be able to critically evaluate and leverage free and/or open-source AI tools, programming libraries and datasets.”	Section 2: Dentistry for individual patients: better understanding for better care “Dental professionals should critically evaluate the certification status of AI products before employing them for patient care.” Section 5: Research “Researchers should adopt existing checklists and reporting standards for AI research, and reviewers and editors should contrast any submissions against these baseline reporting expectations” “Researchers but also the recipients of research, dentists and other decision makers, need to be able to appraise AI research and the developed applications critically” Section 6: Risks and Limitations of AI Risk classification at conceptualization, development and implementation & maintainence stages. Bias and generalisation, accessibility, interoperability, truthfulness	Chapter 3 Applications of artificial intelligence for health; in health care, in health research and drug development; in health systems management and planning, in public health and public health surveillance, and the future of artificial intelligence for health. Chapter 5: Key ethical principles for use of artificial intelligence for health; protect autonomy, promote human well-being, human safety and the public interest, ensure transparency, explainability and intelligibility, foster responsibility and accountability, ensure inclusiveness and quity, promote artificial intelligence that is responsive and sustainable. Chapter 6: Ethical challenges to use of artificial intelligence for health care	Strategy 1: Preparing the country in society, ethics, law and regulations for AI application
CUAI-3: AI Tool Discovery and Utilisation Discover and utilize available AI tools, programming libraries, and/or datasets to enhance the efficiency and quality of human-centric tasks.	1. Explores essential AI tools. 2. Evaluates, and deploys AI tools to enhance the efficiency and quality of human-centric tasks, ensuring responsible and effective use in real-world workflows	Aspect: AI techniques and applications: Application skills (Level 2: Apply) & Creating AI tools (Level 3: Create) “Students are expected to deepen and apply knowledge and skills on data and algorithms to customize existing AI toolkits to create task-based AI tools.”	Section 2: Dentistry for individual patients: better understanding for better care Image analysis, Data synthesis and prediction, Evidence-support planning and conduct therapies, patient interaction. Section 3: Community and Public Health “AI and data-driven tools will enrich and help to expand community and public dental health services.” Section 5: Research“Data-driven technologies including AI will facilitate better information and decision-making on the dental public health level”	Chapter 3 Applications of artificial intelligence for health; in health care, in health research and drug development; in health systems management and planning, in public health and public health surveillance, and the future of artificial intelligence for health. Chapter 5: Key ethical principles for use of artificial intelligence for health	Strategy 5: Promoting application of AI technologies and systems in public and private sectors
2. AI awareness and safety					
CUAI-4: Digital Content Consumption in the AI Era Critically evaluate the accuracy of AI-generated results and remain mindful that any digital content can be AI-generated, potentially lacking transparency and truthfulness.	1. Evaluate the accuracy, reliability, and integrity of digital content, assuming that any content may be AI-generated. 2. Demonstrates skills to verify sources, detect misinformation, and apply judgment before use or dissemination	Aspect: Ethics of AI: Embodied ethics/Safe and responsible use; Human-centred mindset – Citizenship in the AI era	Section 6: Risks and Limitations of AI Risk classification at conceptualization, development and implementation & maintainence stages Bias and generalisation, accessibility, interoperability, truthfulness	Chapter 5: Key ethical principles for use of artificial intelligence for health; protect autonomy, promote human well-being, human safety and the public interest, ensure transparency, explainability and intelligibility, foster responsibility and accountability, ensure inclusiveness and quity, promote artificial intelligence that is responsive and sustainable.	Strategy 1: Society, ethics, law and regulations
CUAI-5: AI-related risk identification and handling Identify AI-related risks and take actions to protect personal and peer safety.	1. Recognise and mitigate risks associated with the use of AI, including but not limited to privacy violations and security breaches. 2. Take action to protect personal, peer, and organisational safety.	Aspect: Ethics of AI: Safe and responsible use; Human-centred mindset: Human accountability “They are aware of the risks of disclosing data privacy and they take measures to ensure that their data are collected, used, shared, archived and deleted only with their deliberate and informed consent.”	Section 6: Risks and Limitations of AI Risk classification at conceptualization, development and implementation & maintainence stages Bias and generalisation, accessibility, interoperability, truthfulness	Chapter 5: Key ethical principles for use of artificial intelligence for health; protect autonomy, promote human well-being, human safety and the public interest, ensure transparency, explainability and intelligibility, foster responsibility and accountability, ensure inclusiveness and quity, promote artificial intelligence that is responsive and sustainable.	Strategy 1: Ethics, law and regulations
3. AI system design					
CUAI-6: AI System Design Design AI systems by selecting appropriate techniques, defining data requirements, and establishing test/feedback metrics.	1. Designs AI workflow/systems by selecting appropriate techniques and identifying the required information to meet task objectives. 2. Ensures the end-to-end lifecycle considers requirements, data strategy, modeling choices, evaluation, deployment/integration, responsible AI, and sustainability/maintenance.	Aspect: AI system design: Problem scoping, Architecture design, Iteration and feedback loops. “Systemic design thinking and comprehensive engineering skills required for problem scoping, design, architecture building, training, testing and optimization of AI systems.”	Section 2: Dentistry for individual patients: better understanding for better care. Section 3: Community and Public Health Section 4: Education and the workforce Section 5: Research	Chapter 4: Law, policies and principles that apply to artificial intelligence for health; Artificial intelligence and human rights, data protection laws and policies, existing laws and policies related to health data, general principles for the development and use of artificial intelligence, principles for use of artificial intelligence for health, bioethics laws and policies, regulatory considerations. Chapter 5: Key ethical principles for use of artificial intelligence for health; protect autonomy, promote human well-being, human safety and the public interest, ensure transparency, explainability and intelligibility, foster responsibility and accountability, ensure inclusiveness and quity, promote artificial intelligence that is responsive and sustainable.	Strategy 2: AI infrastructure and supporting systems. Strategy 4: Technology and innovation development.
CUAI-7: Solution development Develop task-based AI solutions using tools that are relevant to their field of study	1. Develops AI solutions to address specific task requirements by translating needs into implementable designs. 2. Build and validate models or toolchains in human-centric workflows with responsible and safe operation throughout the lifecycle	Aspect: AI system design: Architecture design (Level 2: Apply) and Iteration & feedback (Level 3: Create); AI techniques – Creating AI tools “Cultivate basic methodological knowledge and technical skills to configure a scalable, maintainable and reusable architecture for an AI system.” “Iteration and feedback: improve datasets, reconfigure algorithms and enhance architectures in response to test and feedback.”	Section 2: Dentistry for individual patients: better understanding for better care. Section 3: Community and Public Health. Section 4: Education and the workforce. Section 5: Research. Section 6: Risks and Limitations of AI.	Chapter 5: Key ethical principles for use of artificial intelligence for health; protect autonomy, promote human well-being, human safety and the public interest, ensure transparency, explainability and intelligibility, foster responsibility and accountability, ensure inclusiveness and quity, promote artificial intelligence that is responsive and sustainable. Chapter 7 Building an ethical approach to use of artificial intelligence for health; ethical, transparent design of technologies, impact assessment	Strategy 4: Technology and innovation development for AI
4. Ethics, Governance and Sustainable AI Use					
CUAI-8: Human role in AI. Understand the human role in AI development and identify areas of accountability	1. Understands the role of humans in AI development and utilisation. 2. Identify, assign, and exercise accountability and responsibility between humans and AI.	Aspect: Human-centred mindset: Human agency/Human accountability. “Students are expected to recognize that human accountabilities are the legal obligations of AI creators and AI service providers”	Section 7: Governance. Who’s consensus principles on AI. Implemented governance strategies. “Humans should supervise the AI upstream, and downstream accountability remains with the human user.” “Human agency and oversight, including fundamental rights, human agency and human oversight”	Chapter 5: Key ethical principles for use of artificial intelligence for health; Foster responsibility and accountability	Strategy 1: Society, ethics, law and regulations
CUAI-9: Responsible AI practice. Implement responsible AI practices that adhere to ethical principles and support inclusive, sustainable development.	• Use AI practices that aligned with ethical standards and governance requirements (fairness, privacy, transparency, accountability)	Aspect: Ethics of AI: Embodied ethics, Safe and responsible use, Ethics by design. Lists ethical principles to internalise: Do no harm, Proportionality, Non-discrimination, Sustainability, Human determination, Transparency and explainability, Safe and responsible use, Ethics by design.	Section 7: Governance Who’s consensus principles on AI: protect human autonomy; do no harm; transparency, explainability, intelligibility; responsibility and accountability; inclusiveness and equity; responsive and sustainable AI. Implemented governance strategies: EU Commission 7 requirements for AI application – including human agency oversight, technical robustness and safety, privacy and data governance, transparency, diversity, societal and environmental well-being, and accountability.	Chapter 5: Key ethical principles for use of artificial intelligence for health; protect autonomy, promote human well-being, human safety and the public interest, ensure transparency, explainability and intelligibility, foster responsibility and accountability, ensure inclusiveness and quity, promote artificial intelligence that is responsive and sustainable.	Strategy 1: Society, ethics, law and regulations
CUAI-10: Use of AI for personal growth. Cultivate a lifelong curiosity for learning and utilizing AI to support personal growth.	• Demonstrate self-directed learning, productivity, creativity, and well-being, while reflecting on responsible, sustainable, and balanced AI use	Aspect: Human-centred mindset: Citizenship in the AI era; AI techniques: Application skills. “Students are also expected to develop a desire to continue learning about, and using, AI throughout their lives to support self-actualization.”	Section 4: Education and the workforce.	Chapter 5: Key ethical principles for use of artificial intelligence for health; Promote human well-being, human safety and the public interest and Responsive and sustainable AI.	Strategy 3: Personnel capacity and AI education.

The first theme of Chula AI 10 is AI knowledge. This theme comprises three competencies: CUAI-01) Foundation knowledge of AI, CUAI-02) AI use case evaluation, and CUAI-03) AI tool discovery and utilisation. The foundational knowledge required for proficiency in AI includes a comprehensive understanding of AI and machine learning, their evolving capabilities and inherent limitations, and the essential digital competencies needed to interact effectively with these technologies. With a firm foundation of knowledge, students can then critically evaluate potential AI use cases and select appropriate tools and technologies to address specific tasks or needs (5). This approach acknowledges both the progressive capabilities of AI systems and their inherent limitations, thereby fostering adaptability and sustained engagement in lifelong learning. The second competency pertains to assessing AI use cases. Learners are equipped to assess the feasibility, appropriateness, and ethical implications of implementing AI in specific tasks. In addition to the usage-related dimensions, students must also be able to analyse the advantages, risks, inherent biases, limitations, and financial consequences of the utilised AI tools. After gaining access to AI use cases, learners can explore and deploy AI tools to enhance the efficiency and quality of human-centric tasks.

The second theme of Chula AI 10 focuses on AI awareness and safety. This theme contains two competencies: CUAI-04) Digital content consumption in the AI era and CUAI-05) AI-related risk identification and handling. In the AI era, all digital content can potentially be generated by AI. Therefore, learners’ ability to critically evaluate the accuracy of AI-generated information/data is essential. Further, the competency addresses the ability to recognise and mitigate risks associated with the use of AI, including but not limited to privacy violations and security breaches. Learners are expected to take proactive measures to safeguard personal, peer, and organisational security.

The third theme highlights two competencies in AI system design (CUAI-06) and solution development (CUAI-07). Learners are competent in designing AI systems by selecting appropriate techniques and gathering the required information. This leads to the next competency in the development of AI for addressing solutions to specific tasks. This theme aligns with research and scholarly development at the undergraduate level and is offered as an optional pathway (i.e., elective courses) for students seeking deeper engagement in AI tool development within the context of dentistry.

The last theme highlights the ethics, governance, and sustainability of AI use. Human role in AI (CUAI-08) highlights the competency in understanding the role of humans in AI development and utilisation. Learners must be able to identify the areas of accountability and responsibility between humans and AI. Furthermore, the responsible AI practice competency (CUAI-09) focuses on implementing responsible AI practices aligned with ethical standards and promoting inclusive, sustainable development. The last competency (CUAI-10) describes the use of AI for personal growth. In this regard, learners can cultivate a lifelong curiosity for learning and utilising AI to support personal growth. This theme is integral to shaping desirable graduate attributes and is therefore reinforced throughout the curriculum, from preclinical to clinical levels.

### 4.5 AI content integration in the undergraduate dental curriculum

The conventional undergraduate dental curriculum should be transformed to align with advancements in AI (12, 13). The principles of AI should be imparted to undergraduate dental students in a manner that complements the current dental educational framework. The evolution of AI technologies is exceedingly rapid. Therefore, the curricular content must be structured to equip students with the proficiency needed to understand AI tools that are continually evolving. Curricula should encompass the essential terminology, foundational principles and applications, current AI usage and implementations, and ethical considerations while also fostering an understanding of the limitations and challenges of using AI (5, 12). Integrating these elements into an undergraduate dental curriculum could provide an optimal foundation for future dentists to emphasise patient safety, uphold ethical standards, and promote the responsible application of AI in their professional practice (12).

This section highlights the integration of AI literacy into the undergraduate dental curriculum at the Faculty of Dentistry, Chulalongkorn University, to foster AI literacy in line with the proposed competency framework. To structurally integrate AI literacy into the undergraduate dental curriculum, key content was identified and listed in Table 1. The topics were both horizontally and vertically integrated across the educational stages, from foundational knowledge, use cases, and practical implementation to advanced AI concepts and applications in dentistry. The contents were then assigned to the relevant courses and mapped to the list of competencies from the Chula AI 10 framework, as shown in Supplementary Table 2.

Foundational and introductory knowledge of AI was incorporated into the early stages of undergraduate dental curricula as part of basic science and first-year courses in dentistry. Fundamental understanding of AI, including definitions, concepts, techniques, and terminology, was provided. In this early stage, the AI use case in general education, for example, AI tools practice for searching academic content, was also introduced.

In the preclinical stage, the students would learn more specific AI use cases and applications in dentistry, such as diagnostic imaging, medical examinations and detection, and classification approaches for clinical information. The students will also learn the necessary skills to evaluate and appraise the ethical considerations for AI used in dental education and dental health services through the active discussion of case studies.

Students will learn how to integrate and implement AI-based dental tools into dental practice when they progress to the clinical stage. AI-integrated components are scattered throughout clinical courses, such as AI-powered imaging tools for radiographic interpretation and AI-supported patient management systems. In the last year of the curriculum, students will learn advanced, up-to-date topics in AI in dentistry, governance, and its impact on the dental profession.

In the undergraduate dental curriculum, conducting a research project is a requirement. Therefore, the competencies regarding the AI system design and solution development are expected to be implemented and evaluated through this project. AI utilisation as a tool to perform target tasks in research and development processes is in practice, while tightly adhering to ethical standards of use.

In addition to the core courses listed, students must complete 2 and 6 credits of elective courses in years 3 and 6, respectively. In these elective courses, students can take AI-related courses to deepen their AI literacy in specific dental domains that align with their interests.

## 5 Conclusion

The integration of AI into dental education presents both opportunities and challenges. Dental schools can equip future professionals with the necessary skills to leverage AI effectively and ethically. This proactive approach not only enhances the quality of dental education but also improves patient care in an increasingly technology-driven landscape. Collaboration among educators, practitioners, and technology experts is crucial in shaping the future of AI in dentistry.
