# Supplementary material for: Developing a competency framework for artificial intelligence in undergraduate dental education
Source: Front Dent Med. 2026 Jun 11;7:1849000. doi: 10.3389/fdmed.2026.1849000 (PMC13294060; doi:10.3389/fdmed.2026.1849000)
Supplement: Supplementary file 1 [file Table1.docx]

**Scopus search terms:**

( TITLE-ABS-KEY ( "artificial intelligence" OR "machine learning" OR "deep learning" OR "neural network*" OR "AI" ) AND TITLE-ABS-KEY ( "competenc*" OR "competency framework*" OR "competency-based" OR "learning outcome*" OR "curriculum" OR "curriculum development" ) AND TITLE-ABS-KEY ( "dental education" OR "dental curriculum" OR "dental student*" OR "dental school*" OR "undergraduate dental" OR "predoctoral dental" ) )

**Pubmed search terms:**

( ("artificial intelligence"[MeSH Terms] OR "artificial intelligence"[Text Word] OR "machine learning"[MeSH Terms] OR "machine learning"[Text Word] OR "deep learning"[MeSH Terms] OR "deep learning"[Text Word] OR "neural networks, computer"[MeSH Terms] OR "AI"[Text Word]) ) AND ( ("competency-based education"[MeSH Terms] OR "clinical competence"[MeSH Terms] OR "competenc*"[Text Word] OR "competency framework*"[Text Word] OR "competency-based"[Text Word] OR "learning outcome*"[Text Word] OR "curriculum"[MeSH Terms] OR "curriculum development"[Text Word]) ) AND ( ("education, dental"[MeSH Terms] OR "education, dental, graduate"[MeSH Terms] OR "students, dental"[MeSH Terms] OR "schools, dental"[MeSH Terms] OR "dental education"[Text Word] OR "dental curriculum"[Text Word] OR "dental student*"[Text Word] OR "dental school*"[Text Word] OR "undergraduate dental"[Text Word] OR "predoctoral dental"[Text Word]) )
