# Supplementary material for: Developing a competency framework for artificial intelligence in undergraduate dental education
Source: Front Dent Med. 2026 Jun 11;7:1849000. doi: 10.3389/fdmed.2026.1849000 (PMC13294060; doi:10.3389/fdmed.2026.1849000)
Supplement: Supplementary file 2 [file Table2.docx]

**Table 2.** Implementation details of AI in undergraduate dentistry curriculum at Chulalongkorn University.

| **Year** | **Course** | **Content** | **CUAI-01** | **CUAI-02** | **CUAI-03** | **CUAI-04** | **CUAI-05** | **CUAI-06** | **CUAI-07** | **CUAI-08** | **CUAI-09** | **CUAI-10** |
| --- | --- | --- | --- | --- | --- | --- | --- | --- | --- | --- | --- | --- |
|  |  |  | Fundamental knowledge of AI | AI use case evaluation | AI tool discovery and utilization | Digital content consumption in the AI era | AI-related risk identification and handling | AI system design | AI solution development | Human role in AI | Responsible AI practice | AI usage for personal growth |
| **Year 1** | Lifelong Learning Skills for Dentists | Introduction to AI in everyday life | **•** |  |  |  |  |  |  |  |  | **•** |
|  |  | Evidence-Based Learning: Problem Setting and PICO – AI for Formulating Research Question |  | **•** | **•** |  |  |  |  |  |  |  |
|  |  | Evidence-Based Learning: Information Search, Academic Databases, and Levels of Evidence – AI for Information Search |  |  | **•** | **•** |  |  |  |  |  |  |
|  |  | Evidence-Based Learning: Trustworthiness of Evidence, Dealing with Quantitative and Qualitative Data, Critical Appraisal of Information – AI for Analysing Data |  |  | **•** |  |  |  |  | **•** |  |  |
|  |  | Report Writing: Components of an Academic Report, Academic Writing, Referencing, Plagiarism – AI for Report Writing |  |  | **•** |  |  |  |  | **•** | **•** |  |
|  |  | Presenting Data: Data Handling, Data Summarising, Data Visualisation – AI for Presenting Data |  |  | **•** |  |  |  |  | **•** | **•** |  |
|  |  | Design Thinking and Innovation – AI in Design Thinking |  |  | **•** |  |  | **•** | **•** |  |  |  |
|  |  | Evidence-Based Dentistry Project |  | **•** | **•** | **•** | **•** |  |  | **•** | **•** | **•** |
|  | Physics in Dentistry | Introduction of AI in Dentistry | **•** |  |  |  |  |  |  |  |  |  |
|  | Professional Development 1 | Introduction and overview of AI in dental education | **•** |  |  | **•** |  |  |  |  |  |  |
|  |  | Ethical consideration in AI in dental education |  |  |  |  | **•** |  |  | **•** | **•** |  |
| **Year 2** | Radiology 1 | AI and Digital technology for oral and maxillofacial radiology |  | **•** | **•** |  |  | **•** | **•** |  |  |  |
|  | Professional Development 2 | Case study in ethical consideration of AI used in dental education and healthcare |  |  |  | **•** | **•** |  |  | **•** | **•** |  |
| **Year 3** | Professional Development 3 | Case study in AI implementation in Clinical Dentistry (detection and classification approaches: efficacy and limitation) |  | **•** | **•** | **•** | **•** | **•** | **•** | **•** | **•** | **•** |
|  | Dental Research Methodology | Artificial Intelligence in Dental Research Methodology; Assisting Technology and Ethical Consideration |  | **•** | **•** |  | **•** | **•** | **•** | **•** | **•** | **•** |
|  | Elective courses | (Optional) |  |  | **·** |  | **·** | **·** | **·** |  |  |  |
| **Year 4** | Dental Research Project I | Practice of AI utilization in Research Project |  |  | **·** |  |  | **·** | **·** |  |  | **·** |
| **Year 5** | Dental Research Project II | Practice of AI utilization in Research Project |  |  | **·** |  |  | **·** | **·** |  |  | **·** |
|  | Competency in Radiology | Use case of AI in Dentistry |  | **·** | **·** |  | **·** |  |  | **·** |  |  |
| **Year 6** | Digital Technology in Dentistry | Advanced and update of AI in Dentistry |  | **·** | **·** | **·** |  | **·** | **·** |  |  | **·** |
|  | Elective courses | (Optional) |  |  | **·** |  | **·** | **·** | **·** |  |  |  |
